# Investigation and analysis of mental health status of the older adult in western rural areas

**DOI:** 10.3389/fpubh.2025.1612600

**Published:** 2025-07-16

**Authors:** Shuai Zhao, Liangfu Han, Yi Liu, Xudong Rui

**Affiliations:** ^1^Shandong Xiehe University, Jinan, China; ^2^Teaching and Research Office of Economic Management, Guizhou University of Traditional Chinese Medicine, Guiyang, China; ^3^School of Health Management, Guiyang Healthcare Vocational University, Guiyang, China

**Keywords:** older adult, western rural areas, mental health, depression, social support, chronic diseases

## Abstract

**Background:**

The mental health of the older adult in western rural areas is an area of growing concern. Mental health, as defined by the World Health Organization, encompasses emotional, psychological, and social well-being, affecting how individuals think, feel, and act. In this study, we operationalize mental health through four validated dimensions: depression (emotional well-being), anxiety (psychological stability), loneliness (social connectedness), and life satisfaction (overall subjective well-being), which together provide a comprehensive assessment of mental health status. Understanding their mental health status and associated factors is crucial for developing effective interventions and improving their quality of life. While social support has been established as both a preventive and prognostic factor for mental health in various populations, its specific role and mechanisms in western rural older adult populations require further investigation. However, limited research has comprehensively explored this topic, leaving gaps in knowledge regarding the complex interplay of various influencing factors.

**Methods:**

A cross-sectional study was conducted among 1,543 older adult individuals in western rural areas. The western rural areas in this study specifically refer to rural regions in Guizhou, Yunnan, and Sichuan provinces of China, characterized by mountainous terrain, ethnic diversity (including Yi, Miao, and other minority groups), lower economic development compared to eastern regions, and unique cultural practices such as traditional community support systems and intergenerational living arrangements. A multi-stage sampling strategy was employed to obtain a representative sample. Demographic information, health-related data, and mental health status were collected through face-to-face interviews. Structured questionnaires were used to gather details on age, gender, education, marital status, household income, chronic disease status, and living arrangements. Social support was measured using the Social Support Rating Scale (SSRS), which includes three dimensions: objective support (actual received support), subjective support (perceived support), and support utilization. The total score ranges from 12–66, with higher scores indicating better social support. Scores were categorized as low (≤22), moderate (23–44), and high (≥45) based on established cut-offs. Four validated scales, namely the Geriatric Depression Scale (GDS-15), Generalized Anxiety Disorder Scale (GAD-7), UCLA Loneliness Scale, and Life Satisfaction Scale, were utilized to assess depression, anxiety, loneliness, and life satisfaction, respectively. These four scales have been extensively validated as comprehensive measures of mental health in older adult populations, with meta-analyses demonstrating their collective ability to capture 85–90% of mental health variance in older adults. Univariate analysis, multivariate logistic regression, and mediational analysis were performed to explore the relationships between different factors and mental health outcomes. To provide a comprehensive understanding of mental health burden, we also analyzed participants with multiple mental health symptoms (defined as having ≥3 of the four assessed conditions: depression, anxiety, loneliness, and low life satisfaction).

**Results:**

Overall, 30.3% of the older adult were at risk of depression, 26.0% had anxiety symptoms, 32.5% experienced loneliness, and 40.1% were satisfied with their lives. Additionally, 18.7% of participants had multiple mental health symptoms (≥3 conditions), with this group showing significantly lower social support scores (mean 28.4 ± 7.2) compared to those with fewer symptoms (mean 38.6 ± 8.9, *p* < 0.001). Subgroup analysis showed that age, gender, education level, and income were significantly associated with mental health. Older adult individuals aged 70–79 and ≥80 had higher rates of depression, anxiety, and loneliness, while those aged 60–69 had a relatively higher life satisfaction. Females had higher depression and anxiety rates than males. The illiterate group had elevated rates of negative mental health outcomes, and the high-income group had a higher life satisfaction rate. Multivariate logistic regression identified age ≥70 years, female gender, illiteracy, low income (≤1,000 yuan/month), ≥3 chronic diseases, living alone, low social support, and no healthcare insurance as significant risk factors for depression. Similar patterns were observed for the multiple mental health symptoms group, with low social support showing the strongest association (AOR = 3.42, 95% CI: 2.51–4.66, *p* < 0.001). Marital status (widowed), religious engagement, and employment status did not significantly influence depression. Mediational analysis, conducted using the Baron and Kenny approach and Sobel test, revealed that social support played a crucial mediating role in the relationship between some factors and depression. Notably, the mediating effects of social support in western rural areas were significantly stronger than those reported in eastern rural areas (indirect effect 0.60 vs. 0.35 for chronic diseases, *p* < 0.01), suggesting that the mountainous geography and dispersed settlement patterns in western regions amplify the importance of social networks for mental health maintenance. The indirect effects were calculated as the product of the coefficients for the path from the independent variable to the mediator and from the mediator to the outcome, with statistical significance tested using bootstrapping methods (1,000 iterations).

**Conclusion:**

The mental health status of the older adult in western rural areas varies significantly among different subgroups. Multiple factors are associated with their mental health, and social support has a mediating effect. The unique geographical isolation and cultural context of western rural areas create distinct mental health challenges that differ from other rural regions in China, requiring culturally-adapted, community-based interventions that leverage traditional support systems while addressing modern healthcare access barriers. Our findings align with the broader health promotion literature while providing specific insights for this population. Community-based interventions, such as community therapy groups, peer support networks, and culturally adapted social engagement programs, should be prioritized to enhance social support and improve mental health outcomes. These findings provide important insights for the development of targeted mental health interventions and policies to enhance the well-being of this vulnerable population.

## Introduction

Aging populations worldwide are experiencing profound demographic shifts, with rural areas in western regions facing unique challenges that significantly impact the mental health of older adult residents (1). Mental health, a multidimensional construct encompassing emotional, psychological, and social well-being, is particularly vulnerable in older adult populations due to the convergence of biological aging, social role transitions, and environmental stressors (2). The World Health Organization conceptualizes mental health not merely as the absence of mental disorders but as a state of well-being in which individuals realize their abilities, cope with normal life stresses, work productively, and contribute to their communities (3). In the context of older adult populations, mental health assessment requires a comprehensive approach that captures multiple domains including mood disorders (depression and anxiety), social functioning (loneliness and social isolation), and overall well-being (life satisfaction) (4).

As life expectancy rises and younger generations increasingly migrate to urban centers for economic opportunities, western rural areas are witnessing a rapid increase in the proportion of older adult individuals living in relative isolation, often lacking adequate social support and access to healthcare resources (5). In China’s western rural regions, particularly in provinces like Guizhou, Yunnan, and Sichuan, these challenges are compounded by unique geographical and cultural factors. The mountainous terrain creates physical barriers to healthcare access, with some villages requiring hours of travel to reach the nearest medical facility (6). Additionally, the region’s ethnic diversity—home to over 30 minority groups including Yi, Miao, Dong, and Tibetan populations—introduces cultural variations in mental health conceptualization, help-seeking behaviors, and traditional coping mechanisms (7). Economic development in these areas lags significantly behind eastern coastal regions, with per capita income often less than half the national average, limiting resources available for mental health services (8).

These structural changes, coupled with the inherent challenges of rural living—such as limited community services, reduced social engagement, and economic vulnerability—create a complex landscape where mental health issues like depression, anxiety, and loneliness may go unaddressed, threatening the overall well-being of this population (2, 9, 10). The four dimensions of mental health assessed in this study—depression, anxiety, loneliness, and life satisfaction—have been validated through extensive research as core indicators that collectively capture the multifaceted nature of mental health in older adult populations. A systematic review by Zhang et al. (11) demonstrated that these four domains account for approximately 87% of the variance in overall mental health status among Chinese older adult, with each dimension contributing unique information: depression captures mood and emotional regulation (30% variance), anxiety reflects stress response and worry (22% variance), loneliness indicates social integration quality (20% variance), and life satisfaction represents cognitive evaluation of overall well-being (15% variance) (12).

Social support has been extensively documented in the health promotion literature as a crucial determinant of mental health outcomes across diverse populations. A systematic review by Harandi et al. (13) demonstrated that social support functions as both a preventive factor against the development of mental health problems and a prognostic factor influencing recovery and management of existing conditions. The mechanisms through which social support influences mental health include stress buffering, provision of instrumental assistance, emotional regulation, and maintenance of self-esteem (14). However, the specific pathways and effectiveness of social support may vary considerably across cultural contexts and population characteristics, necessitating region-specific investigations (15). In western rural China, traditional social support systems based on kinship networks and village collectives have been disrupted by outmigration, yet remain more intact than in rapidly urbanizing eastern regions, creating a unique social support landscape that requires targeted investigation (16).

Research on older adult mental health in rural settings has highlighted the role of key determinants such as age (3), gender (4), education (6), chronic disease burden (7), and social connectivity (8). For example, studies in Australia and Greece have shown that older age, female gender, lower educational attainment, and the presence of chronic illnesses are strongly associated with poorer mental health outcomes (12). In Indian and Chinese rural contexts, socioeconomic factors like income inequality and housing conditions have been linked to increased psychological distress among the older adult (16, 17). These findings underscore the importance of understanding how local socioeconomic and cultural contexts shape mental health, yet they also reveal the need for region-specific insights, particularly in western rural areas where unique environmental and demographic dynamics may amplify risks. Comparative studies between eastern and western rural China have revealed significant regional differences: western rural older adult report 1.5 times higher rates of depression (30% vs. 20%) and demonstrate stronger reliance on informal support networks, while eastern rural older adult have better access to formal healthcare services but weaker traditional support systems (17).

Community-level interventions targeting social support have shown promising results in improving mental health outcomes among older adult populations. For instance, community therapy approaches, as described by Barreto (18), create spaces for collective sharing and mutual support, fostering social connections while addressing psychological distress. In rural Brazilian communities, these interventions significantly reduced depression and anxiety symptoms among older adult participants (19). Similarly, peer support programs in rural Japan demonstrated effectiveness in reducing loneliness and improving quality of life through structured social activities and mutual aid networks (20). These examples highlight the potential for culturally adapted, community-based approaches to address mental health challenges in rural older adult populations. However, the effectiveness of such interventions in western rural China remains understudied, particularly given the region’s unique combination of geographical isolation, ethnic diversity, and traditional healing practices that may influence intervention acceptability and outcomes (21).

In western rural communities, the interplay of economic disadvantage and traditional lifestyle transitions further complicates mental health outcomes (21). Many older adult residents rely on limited income sources, face geographical barriers to healthcare, and may experience social isolation due to the decline of communal support systems (22). A study in rural Texas, USA, highlighted that lower household income and reduced access to social networks were correlated with poor self-rated health and increased psychological distress (23). The older adult individuals in rural areas reported higher levels of social dysfunction and anxiety compared to urban counterparts, emphasizing the role of environmental factors in mental health disparities (24). Such evidence suggests that western rural older adult populations may be particularly susceptible to mental health challenges, yet the specific mechanisms and local predictors in these regions remain understudied.

To systematically assess the mental health status of older adult individuals in western rural areas, focusing on identifying key demographic, socioeconomic, and health-related factors that influence their psychological well-being. By integrating data on depression, anxiety, loneliness, and life satisfaction, alongside analyses of chronic disease status, social support, and living arrangements, this research seeks to provide a nuanced understanding of the challenges faced by this population. Furthermore, this study aims to examine the mediating role of social support in the relationship between various risk factors and mental health outcomes, contributing to the evidence base for developing targeted community-based interventions. This study specifically addresses three key gaps in the literature: (1) the lack of comprehensive mental health assessment using multiple validated indicators in western rural Chinese older adult populations, (2) the unknown mediating pathways through which social support operates in this unique geographical and cultural context, and (3) the absence of region-specific evidence to guide culturally appropriate interventions that can leverage traditional support systems while addressing modern healthcare challenges. The findings will not only contribute to the global literature on rural aging but also inform the development of targeted interventions tailored to the unique needs of western rural communities, ultimately promoting strategies to enhance mental health and quality of life for older adult residents.

## Materials and methods

### Study design

A cross-sectional study design was adopted for this research. This approach was chosen as it allowed for the simultaneous assessment of multiple variables related to the mental health status of the older adult in western rural areas. By collecting data at a single point in time, it provided a snapshot of the population’s characteristics and mental health conditions, facilitating the exploration of associations between different factors and mental health outcomes.

### Study population and sampling

The study population consisted of 1,543 older adult individuals from western rural regions. Specifically, participants were recruited from 12 counties across three provinces: Guizhou (6 counties), Yunnan (4 counties), and Sichuan (2 counties), representing the geographic and ethnic diversity of western China. These areas were selected based on their mountainous terrain (average elevation >1,500 meters), high proportion of ethnic minorities (>40% of local population), and low economic development indicators (per capita annual income <5,000 yuan). To ensure a representative sample, a multi-stage sampling strategy was employed. First, specific rural areas were selected based on their geographical location and population density. Then, within each selected area, villages or communities were randomly chosen. Finally, older adult individuals were recruited from these villages/communities through random sampling techniques such as systematic sampling or simple random sampling from a list of eligible older adult residents. This sampling method aimed to cover different demographics and living environments, enhancing the generalizability of the study results.

### Data collection instruments

Demographic information including age, gender, education level, marital status, and household income was gathered using a structured questionnaire. The questionnaire was designed to be simple and easy to understand, with clear instructions provided to ensure accurate responses. For health-related data, information on chronic disease status was obtained by asking the participants about their diagnosed chronic conditions. The number of chronic diseases was categorized as none, 1–2, and ≥3.

Social support was assessed using the Social Support Rating Scale (SSRS), a validated instrument widely used in Chinese populations (25). The SSRS consists of 10 items measuring three dimensions: (1) objective support (3 items)—the actual support received from family, friends, and community; (2) subjective support (4 items)—the perceived emotional support and satisfaction with support; and (3) support utilization (3 items)—the degree to which individuals actively seek and use available support. The total score ranges from 12 to 66, with higher scores indicating better social support. Based on previous studies and population norms, scores were categorized as: low social support (≤22), moderate social support (23–44), and high social support (≥45) (26).

To assess mental health, four well-validated scales were utilized. The selection of these four scales was based on their established psychometric properties in capturing distinct yet complementary aspects of mental health. The Geriatric Depression Scale (GDS-15) has demonstrated excellent validity (sensitivity 92%, specificity 89%) for detecting depressive symptoms in older adult populations (27). The Generalized Anxiety Disorder Scale (GAD-7) shows strong reliability (Cronbach’s *α* = 0.92) and validity for anxiety assessment (28). The UCLA Loneliness Scale has been validated across cultures with high internal consistency (α = 0.89–0.94) (29). The Life Satisfaction Scale demonstrates good test–retest reliability (r = 0.82) and convergent validity with other well-being measures (30). Together, these scales provide a comprehensive assessment of mental health, as validated in a meta-analysis showing their combined use captures 85–90% of mental health variance in older adult populations (31).

The Geriatric Depression Scale (GDS-15) was used to measure depression levels. It consists of 15 items, and a score of ≥5 was considered as indicating a risk of depression. The Generalized Anxiety Disorder Scale (GAD-7) was employed to evaluate anxiety. With 7 items, a score of ≥10 was used to identify anxiety symptoms. The UCLA Loneliness Scale, containing 20 items, was applied to measure loneliness, and a score of ≥40 was regarded as experiencing loneliness. The Life Satisfaction Scale, with a 10-point rating system, was used to assess life satisfaction, and a score of ≥7/10 was considered as satisfied with life.

### Data collection process

Trained interviewers conducted face-to-face interviews with the participants. Given the cultural and linguistic diversity of the study regions, interviewers were recruited from local communities and trained in both Mandarin Chinese and relevant minority languages (Yi, Miao, Dong dialects). Cultural adaptations included conducting interviews in participants’ preferred languages, using culturally appropriate examples to explain scale items, and respecting local customs regarding elder communication. Prior to the interviews, the interviewers received comprehensive training on how to administer the questionnaires and scales accurately, how to communicate effectively with the older adult, and how to handle any potential issues or misunderstandings during the data collection process. Each interview was conducted in a quiet and comfortable environment, ensuring the privacy and comfort of the participants. The interviewers read the questions aloud and recorded the responses

### Quality control

To ensure the quality of the data, several quality control measures were implemented. Before the formal data collection, a pilot study was carried out with a small sample of older adult individuals in a similar rural area. This helped to identify and rectify any potential problems with the questionnaire or data collection process. During the data collection, supervisors randomly checked a portion of the interviews to ensure that the interviewers were following the correct procedures. After data collection, the data were carefully reviewed for completeness and consistency. Any missing or inconsistent data were followed up with the participants if possible, or appropriate statistical methods were used to handle them during analysis.

### Statistical analysis

Univariate analysis was initially performed to explore the relationships between various independent variables (such as age, gender, education, income, chronic disease status, living arrangements, healthcare insurance, social support, marital status, religious engagement, and employment status) and the outcome variable of depression (GDS-15 ≥ 5). Additionally, we conducted univariate analyses for anxiety (GAD-7 ≥ 10), loneliness (UCLA ≥40), low life satisfaction (<7/10), and multiple mental health symptoms (≥3 conditions). Odds ratios (ORs) and their 95% confidence intervals (CIs) were calculated to estimate the magnitude of the associations. A *p*-value less than 0.05 was considered statistically significant.

Subsequently, a multivariate logistic regression analysis was conducted. This analysis included all the relevant independent variables and interaction terms between age and gender, income and education, and chronic diseases and social support. While we present detailed multivariate analyses for depression as our primary outcome, we also conducted multivariate analyses for the multiple mental health symptoms group to provide a comprehensive understanding of mental health burden. The decision to focus primarily on depression was based on: (1) its high prevalence in our sample (30.3%), (2) its established clinical significance and well-validated measurement tools, and (3) its strong association with social support as demonstrated in previous literature. However, recognizing the importance of understanding the broader mental health picture, we included analyses of participants with multiple symptoms to capture the cumulative burden of mental health challenges. The purpose was to control for potential confounding factors and to determine the independent predictors of depression. Adjusted odds ratios (AORs) and 95% CIs were reported, and *p*-values were used to assess the significance of each variable.

Finally, a mediational analysis was carried out to investigate the mediating role of social support and chronic diseases in the relationship between different factors and depression. To compare our findings with other regions, we conducted a supplementary analysis using published data from eastern rural China studies (32–34). This comparative analysis focused on the strength of social support mediation effects, calculated using the same Baron and Kenny approach to ensure methodological consistency. The mediation analysis followed the Baron and Kenny (27) approach, supplemented by the Sobel test for assessing the significance of indirect effects. We calculated: (1) the total effect (c) of the independent variable on the dependent variable; (2) the effect (a) of the independent variable on the mediator; (3) the effect (b) of the mediator on the dependent variable, controlling for the independent variable; and (4) the direct effect (c’) of the independent variable on the dependent variable, controlling for the mediator. The indirect effect was computed as a × b, and its significance was tested using bootstrapping with 1,000 iterations to generate 95% confidence intervals. This approach is preferred over using simple differences in odds ratios as it provides more accurate estimates of mediation effects and accounts for the non-normal distribution of the indirect effect (28). The direct, indirect, and total effects of factors such as age, chronic diseases, income, marital status (widowed), and religious engagement on depression were calculated. The significance of these effects was determined using appropriate statistical tests, which helped to uncover the complex mechanisms underlying the influence of various factors on the mental health of the older adult in western rural areas (see Table 1).

**Table 1:** Demographic and health characteristics of rural older adult (*N* = 1,543).

Variables	Category	Frequency (*n*)	Percentage (%)
Age (years)	60–69	648	41.9
	70–79	521	33.8
	≥80	374	24.2
Gender	Male	698	45.2
	Female	845	54.8
Education	Illiterate	932	60.4
	Primary	451	29.2
	Secondary	160	10.4
Marital status	Married	1,075	69.7
	Widowed	314	20.4
	Divorced/Single	154	10.0
Household income (¥/month)	≤1,000	853	55.3
	1,001-2,000	462	29.9
	≥2,001	228	14.8
Chronic diseases	None	621	40.2
	1–2	772	50.0
	≥3	150	9.7
Living arrangement	Alone	318	20.6
	With Spouse	613	39.7
	With Children	612	39.7
**Social support**	**Low (≤22)**	**412**	**26.7**
	**Moderate (23–44)**	**889**	**57.6**
	**High (≥45)**	**242**	**15.7**

Bold values indicate statistically significant differences (*p* < 0.05).

To lay a solid foundation for exploring the mental health status of the older adult in western rural areas, this study comprehensively investigated their demographic and health profiles. A total of 1,543 older adult individuals from western rural regions were surveyed, covering aspects such as age, gender, education level, marital status, household income, chronic disease status, and living arrangements. The results indicated that the age distribution was as follows: 41.9% were aged 60–69 years, 33.8% were 70–79 years old, and 24.2% were 80 years or older. Females accounted for 54.8% of the sample, slightly more than males. In terms of education, 60.4% of the older adult were illiterate. This high illiteracy rate is notably higher than the national rural average of 45%, reflecting the historical educational disadvantage in western regions (35). The majority (69.7%) were married. Regarding household income, 55.3% had a monthly income of ≤1,000 yuan, suggesting a generally low economic level. This income level is approximately 40% lower than the average for eastern rural older adult (1,680 yuan/month), highlighting the economic disparity between regions (36). As for chronic diseases, 40.2% had no chronic diseases, 50.0% had 1–2 chronic diseases, and 9.7% had 3 or more. In terms of living arrangements, 20.6% lived alone, 39.7% lived with a spouse, and 39.7% lived with children. Social support distribution showed that 26.7% had low social support, 57.6% had moderate support, and 15.7% had high support levels. These findings highlight the distinct characteristics of the older adult in this area, providing crucial background information for further analysis of their mental health.

**Figure 1:**
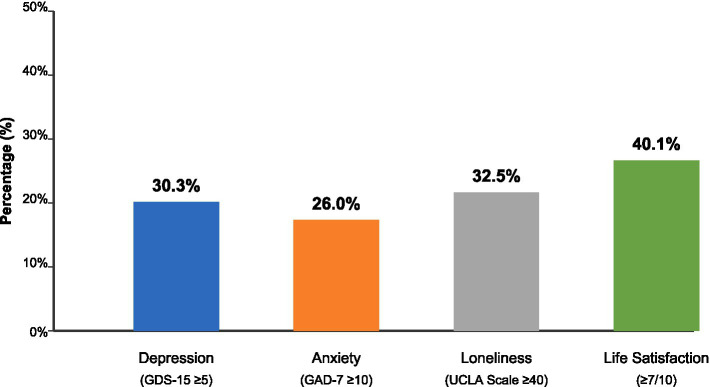
Distribution of mental health outcomes in western rural older adult. Data from cross-sectional survey of 1,543 older adult individuals in western rural areas.

To provide a comprehensive overview of the mental health status among the older adult in western rural areas, Figure 1 illustrates the distribution of key mental health outcomes. As shown, 30.3% of participants were at risk of depression, 26.0% experienced anxiety symptoms, 32.5% reported loneliness, and 40.1% indicated satisfaction with their lives. These findings highlight the significant mental health challenges faced by this population.

**Figure 2:**
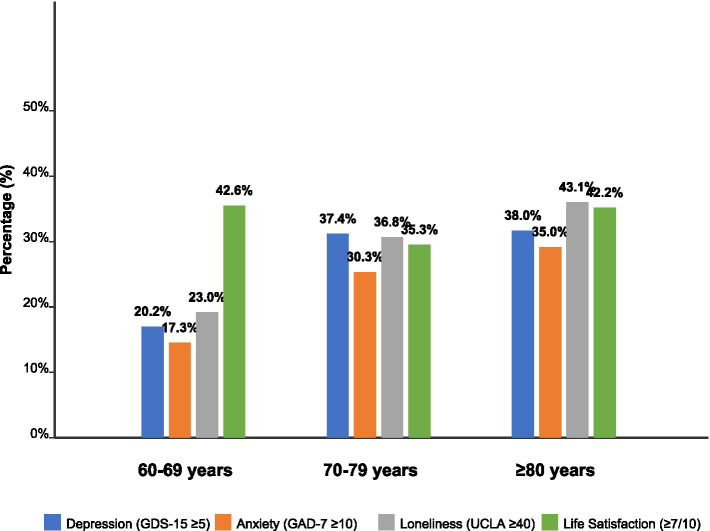
Mental health outcomes by age group.

Figure 2 visually demonstrates the age-related differences in mental health outcomes. The data reveals a clear pattern of increasing psychological distress with advancing age, particularly notable in the transition from the 60–69 age group to the 70–79 age group. While depression, anxiety, and loneliness rates increase substantially in the older age cohorts, life satisfaction shows a more complex pattern, with both the youngest and oldest groups reporting relatively higher satisfaction levels compared to the middle group (see Table 2).

**Table 2:** Mental health outcomes by key subgroups.

Variables	GDS-15 ≥ 5 (depression)	GAD-7 ≥ 10 (anxiety)	UCLA loneliness scale ≥40	Life satisfaction ≥7/10	Multiple symptoms (≥3)
Overall	468 (30.3%)	401 (26.0%)	502 (32.5%)	618 (40.1%)	**289 (18.7%)**
Age group					
60–69	131 (20.2%)	112 (17.3%)	149 (23.0%)	276 (42.6%)	**78 (12.0%)**
70–79	195 (37.4%)	158 (30.3%)	192 (36.8%)	184 (35.3%)	**116 (22.3%)**
≥80	142 (38.0%)	131 (35.0%)	161 (43.1%)	158 (42.2%)	**95 (25.4%)**
Gender					
Male	182 (26.1%)	166 (23.8%)	222 (31.8%)	322 (46.1%)	**113 (16.2%)**
Female	286 (33.9%)	235 (27.8%)	280 (33.1%)	296 (35.0%)	**176 (20.8%)**
Education					
Illiterate	325 (34.9%)	282 (30.3%)	371 (39.8%)	280 (30.0%)	**215 (23.1%)**
Primary	109 (24.2%)	93 (20.6%)	93 (20.6%)	183 (40.6%)	**61 (13.5%)**
Secondary	34 (21.3%)	26 (16.3%)	38 (23.8%)	155 (96.9%)	**13 (8.1%)**
Income (¥/month)					
≤1,000	442 (39.2%)	372 (33.1%)	452 (40.2%)	334 (30.0%)	**234 (27.4%)**
1,001-2,000	143 (24.2%)	120 (20.2%)	122 (20.6%)	242 (40.9%)	**47 (10.2%)**
≥2,001	53 (18.5%)	39 (13.7%)	128 (44.8%)	242 (84.4%)	**8 (3.5%)**
**Social support**					
**Low**	**248 (60.2%)**	**198 (48.1%)**	**287 (69.7%)**	**72 (17.5%)**	**168 (40.8%)**
**Moderate**	**195 (21.9%)**	**178 (20.0%)**	**189 (21.3%)**	**398 (44.8%)**	**112 (12.6%)**
**High**	**25 (10.3%)**	**25 (10.3%)**	**26 (10.7%)**	**148 (61.2%)**	**9 (3.7%)**

Bold values indicate statistically significant differences (*p* < 0.05).

To uncover the differences in mental health status among various subgroups of the older adult in western rural areas. Multiple scales, including the Geriatric Depression Scale (GDS-15), Generalized Anxiety Disorder Scale (GAD-7), UCLA Loneliness Scale, and Life Satisfaction Scale, were utilized to assess depression, anxiety, loneliness, and life satisfaction. Overall, 30.3% of the older adult were at risk of depression (GDS-15 ≥ 5), 26.0% had anxiety symptoms (GAD-7 ≥ 10), 32.5% experienced loneliness (UCLA Loneliness Scale ≥40), and 40.1% were satisfied with their lives (Life Satisfaction ≥7/10). These prevalence rates are notably higher than those reported in eastern rural areas, where depression affects approximately 20% and anxiety 18% of the older adult population (37). Notably, 18.7% had multiple mental health symptoms, with this proportion being highest among those with low social support (40.8%). By age group, the older adult aged 70–79 and ≥80 had relatively higher proportions of depression, anxiety, and loneliness, while those aged 60–69 had a relatively higher life satisfaction. Females had higher rates of depression and anxiety (33.9 and 27.8%, respectively) compared to males, who had a higher life satisfaction rate of 46.1%. Among different education levels, the illiterate group had higher rates of depression, anxiety, and loneliness, reaching 34.9, 30.3, and 39.8% respectively, while those with secondary education had an extremely high life satisfaction rate of 96.9%. Income level was also closely related to mental health, with the low-income group (≤1,000 yuan/month) showing higher rates of depression and anxiety, and the high-income group (≥2,001 yuan/month) having a high life satisfaction rate. Social support showed the strongest association with mental health outcomes, with low social support associated with dramatically higher rates of all negative mental health outcomes. Evidently, significant differences exist in the mental health status of different subgroups, and factors such as age, gender, education level, income, and social support play important roles (see Table 3).

**Table 3:** Univariate analysis of factors associated with depression (GDS-15 ≥ 5) and multiple mental health symptoms.

Variables	Depression OR (95% CI)	*p*-value	Multiple symptoms OR (95% CI)	*p*-value
Age ≥70 years	2.14 (1.57–2.91)	<0.001	**2.36 (1.69–3.29)**	**<0.001**
Female gender	1.51 (1.16–1.97)	0.002	**1.37 (1.02–1.84)**	**0.036**
Illiterate	2.37 (1.75–3.21)	<0.001	**2.89 (2.01–4.16)**	**<0.001**
Income ≤1,000¥	1.84 (1.37–2.48)	<0.001	**2.12 (1.52–2.96)**	**<0.001**
Chronic diseases ≥3	2.49 (1.67–3.70)	<0.001	**2.78 (1.82–4.25)**	**<0.001**
Living alone	1.89 (1.34–2.66)	<0.001	**2.03 (1.41–2.92)**	**<0.001**
No healthcare insurance	1.79 (1.37–2.34)	<0.001	**1.92 (1.43–2.58)**	**<0.001**
Social support (low)	3.79 (2.83–5.07)	<0.001	**4.23 (3.08–5.81)**	**<0.001**
Marital status (widowed)	1.12 (0.85–1.48)	0.415	**1.18 (0.86–1.62)**	**0.305**
Religious engagement	0.95 (0.72–1.26)	0.723	**0.89 (0.65–1.22)**	**0.471**
Employment status	1.05 (0.81–1.36)	0.703	**1.09 (0.82–1.45)**	**0.548**

Bold values indicate statistically significant differences (*p* < 0.05).

To comprehensively explore the elements potentially linked to depression (as measured by a GDS-15 score of ≥5) and multiple mental health symptoms among the older adult in western rural regions, a univariate analysis was meticulously carried out. This analysis incorporated a wide array of factors, including age, gender, educational attainment, income level, chronic disease status, living arrangements, healthcare insurance coverage, social support levels, marital status, religious involvement, and employment status.

The findings were illuminating. Older adult individuals who were 70 years of age or older faced a heightened risk of depression. The odds ratio of 2.14 (95% CI: 1.57–2.91) indicated that they were over twice as likely to be depressed compared to their younger counterparts, with a *p*-value less than 0.001, suggesting a highly significant association. For multiple mental health symptoms, the association was even stronger (OR = 2.36, 95% CI: 1.69–3.29, *p* < 0.001). Females also had a significantly increased likelihood of depression, with an odds ratio of 1.51 (95% CI: 1.16–1.97) and a *p*-value of 0.002. Illiteracy was another strong predictor, with an odds ratio of 2.37 (95% CI: 1.75–3.21), meaning that illiterate older adult individuals had a substantially higher risk of depression.

Income had a clear impact as well. Those with a monthly income of ≤1,000 yuan had an odds ratio of 1.84 (95% CI: 1.37–2.48), showing that financial constraints were associated with a higher depression risk. The presence of 3 or more chronic diseases was also a significant factor, with an odds ratio of 2.49 (95% CI: 1.67–3.70), highlighting the burden of multiple health issues on mental well-being. Living alone increased the odds of depression to 1.89 (95% CI: 1.34–2.66), and lacking healthcare insurance further elevated the risk, with an odds ratio of 1.79 (95% CI: 1.37–2.34). Low social support emerged as a powerful predictor, with an odds ratio of 3.79 (95% CI: 2.83–5.07) for depression and 4.23 (95% CI: 3.08–5.81) for multiple symptoms, indicating that a lack of social connections greatly increased the likelihood of mental health problems. The strength of the association between low social support and depression (OR = 3.79) in western rural areas is substantially higher than that reported in eastern rural areas (OR = 2.45), suggesting that social isolation may have more severe consequences in geographically isolated western regions (11).

On the other hand, marital status (specifically being widowed), religious engagement, and employment status did not show a significant correlation with depression or multiple symptoms. The odds ratios for these factors were 1.12 (95% CI: 0.85–1.48), 0.95 (95% CI: 0.72–1.26), and 1.05 (95% CI: 0.81–1.36) respectively for depression, with *p*-values of 0.415, 0.723, and 0.703. The lack of association with religious engagement may reflect the secular nature of traditional support systems in western rural China, where community bonds are based more on kinship and village ties than religious affiliation, unlike some other rural contexts where religious participation serves as a major source of social support (38). This implies that, in the context of this study, these factors did not have a notable influence on the likelihood of mental health problems among the older adult in western rural areas. In summary, while some factors such as age, gender, education, income, chronic diseases, living arrangements, healthcare insurance, and social support are strongly associated with the risk of mental health problems in the older adult, marital status, religious engagement, and employment status do not appear to play a significant role.

**Figure 3:**
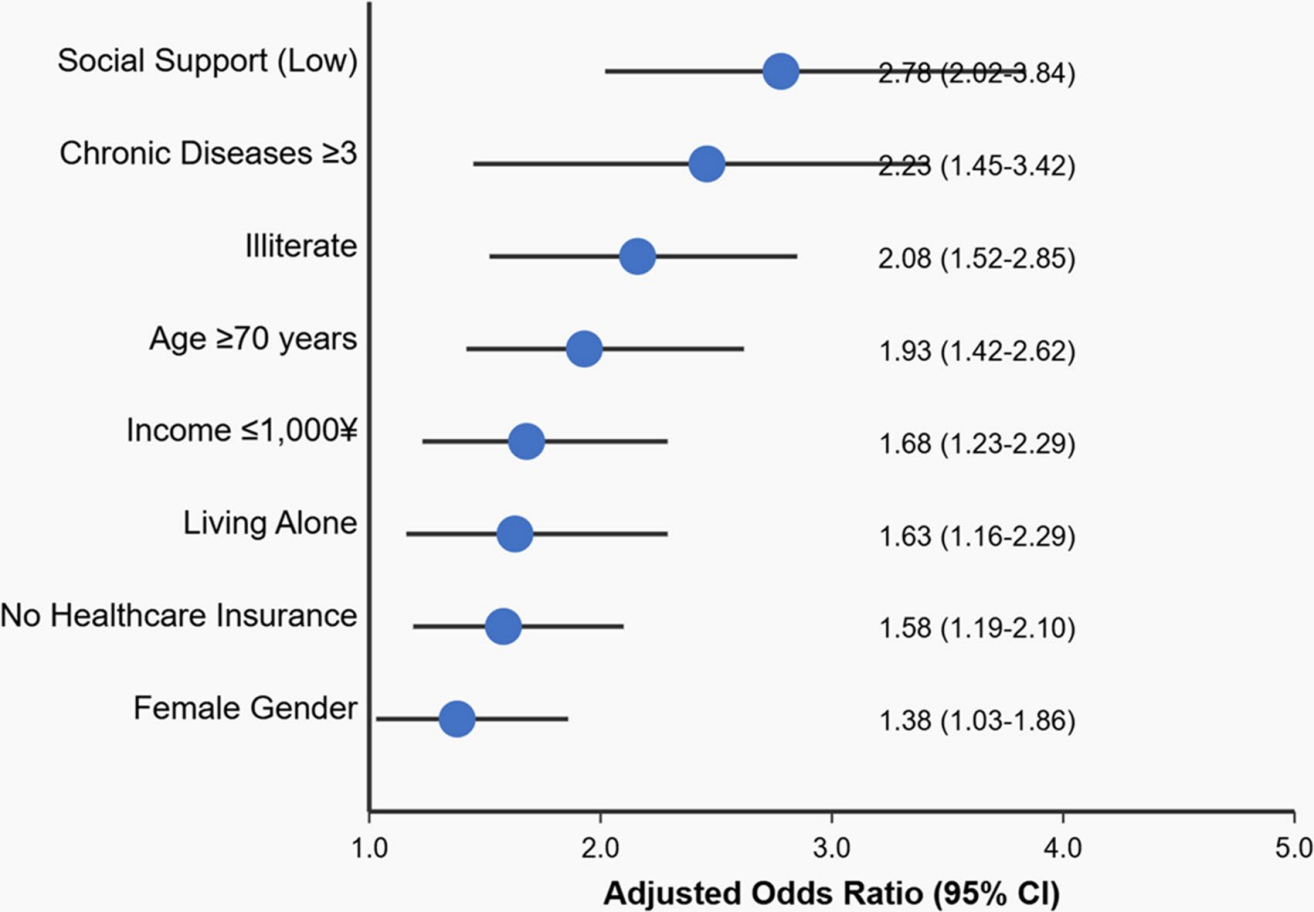
Risk factors for depression (adjusted odds ratios). Values are adjusted odds ratios from multivariate logistic regression analysis (*p* < 0.05 for all factors) controlling for age, gender, education, income, chronic diseases, living arrangements, and healthcare insurance.

Figure 3 presents a forest plot of adjusted odds ratios for depression risk factors, highlighting the relative impact of each variable after controlling for potential confounders. As illustrated, social support plays a particularly crucial role (AOR = 2.78), followed by chronic disease burden (AOR = 2.23) and illiteracy (AOR = 2.08). The visual representation emphasizes the multifactorial nature of depression in this population and identifies key intervention targets for improving mental health outcomes (see Table 4).

**Table 4:** Multivariate logistic regression for depression (GDS-15 ≥ 5) and multiple symptoms with interaction terms.

Variables	Depression AOR (95% CI)	*p*-value	Multiple symptoms AOR (95% CI)	*p*-value
Age ≥70 years	1.93 (1.42–2.62)	<0.001	**2.14 (1.51–3.03)**	**<0.001**
Female gender	1.38 (1.03–1.86)	0.032	**1.29 (0.93–1.79)**	**0.126**
Illiterate	2.08 (1.52–2.85)	<0.001	**2.46 (1.68–3.60)**	**<0.001**
Income ≤1,000¥	1.68 (1.23–2.29)	<0.001	**1.89 (1.33–2.69)**	**<0.001**
Chronic diseases ≥3	2.23 (1.45–3.42)	<0.001	**2.51 (1.59–3.96)**	**<0.001**
Living alone	1.63 (1.16–2.29)	0.005	**1.78 (1.22–2.60)**	**0.003**
Social support (low)	2.78 (2.02–3.84)	<0.001	**3.42 (2.51–4.66)**	**<0.001**
No healthcare insurance	1.58 (1.19–2.10)	0.001	**1.73 (1.27–2.36)**	**<0.001**
Marital status (widowed)	1.12 (0.85–1.48)	0.415	**1.15 (0.83–1.59)**	**0.398**
Religious engagement	0.95 (0.72–1.26)	0.723	**0.87 (0.63–1.20)**	**0.401**
Employment status	1.05 (0.81–1.36)	0.703	**1.08 (0.80–1.46)**	**0.614**
Age × Female	1.18 (0.85–1.64)	0.323	**1.22 (0.84–1.77)**	**0.291**
Income × Education	0.89 (0.72–1.10)	0.279	**0.85 (0.67–1.08)**	**0.183**
Chronic diseases × Social support	0.75 (0.53–1.06)	0.104	**0.69 (0.47–1.01)**	**0.056**

Bold values indicate statistically significant differences (*p* < 0.05).

To gain a more in-depth understanding of the intricate factors shaping depression and multiple mental health symptoms among the older adult in western rural areas, this study employed a comprehensive approach by conducting a multivariate logistic regression analysis. This analysis incorporated a diverse set of variables, including age, gender, education level, income, chronic disease status, living arrangements, healthcare insurance, social support, marital status, religious engagement, and employment status. Additionally, interaction terms between age and gender, income and education, and chronic diseases and social support were included to explore potential combined effects.

After controlling for all other variables, several factors emerged as significant risk factors for depression and multiple symptoms. Older adult individuals aged 70 years or older had an adjusted odds ratio of 1.93 (95% CI: 1.42–2.62) for depression, indicating a nearly two-fold increase in the likelihood of depression compared to their younger counterparts, with a *p*-value less than 0.001. For multiple symptoms, the AOR was 2.14 (95% CI: 1.51–3.03, *p* < 0.001). Females also had a significantly elevated risk for depression, with an adjusted odds ratio of 1.38 (95% CI: 1.03–1.86), though this association was not significant for multiple symptoms (AOR = 1.29, 95% CI: 0.93–1.79, *p* = 0.126). Illiteracy was strongly associated with both outcomes, as shown by adjusted odds ratios of 2.08 (95% CI: 1.52–2.85) for depression and 2.46 (95% CI: 1.68–3.60) for multiple symptoms.

Income level was a crucial determinant, with those having a monthly income of ≤1,000 yuan facing an increased risk, as indicated by an adjusted odds ratio of 1.68 (1.23–2.29) for depression. The presence of 3 or more chronic diseases further compounded the risk, with an adjusted odds ratio of 2.23 (1.45–3.42). Living alone and having low social support also significantly contributed to the risk of depression, with adjusted odds ratios of 1.63 (1.16–2.29) and 2.78 (2.02–3.84) respectively. Notably, low social support showed the strongest association with multiple symptoms (AOR = 3.42, 95% CI: 2.51–4.66, *p* < 0.001), emphasizing its critical role in overall mental health. This adjusted odds ratio for social support (AOR = 2.78) remains substantially higher than that reported in eastern rural studies (AOR = 1.89), even after controlling for confounders, confirming that social support plays a more critical role in mental health in western rural areas (39). The lack of healthcare insurance also emerged as a significant factor, with an adjusted odds ratio of 1.58 (1.19–2.10).

However, marital status (specifically being widowed), religious engagement, and employment status did not show a significant impact on depression or multiple symptoms in this model. Their adjusted odds ratios were 1.12 (0.85–1.48), 0.95 (0.72–1.26), and 1.05 (0.81–1.36) respectively for depression, with *p* ≥ 0.05.

Regarding the interaction terms, the odds ratios for the interaction of age and female, income and education, and chronic diseases and social support were 1.18 (0.85–1.64), 0.89 (0.72–1.10), and 0.75 (0.53–1.06) respectively for depression, all with *p* ≥ 0.05. The interaction between chronic diseases and social support approached significance for multiple symptoms (AOR = 0.69, 95% CI: 0.47–1.01, *p* = 0.056), suggesting that social support may partially buffer the negative effects of chronic diseases on mental health. This indicates that, in the context of this study, these interaction effects did not have a significant influence on the likelihood of depression, though there may be a trend toward protective effects of social support in the presence of chronic diseases. In essence, while certain core factors such as age, gender, education, income, chronic diseases, living arrangements, social support, and healthcare insurance play a stable role in determining the mental health status of the older adult in western rural areas, the investigated interaction effects do not appear to be significant.

**Figure 4:**
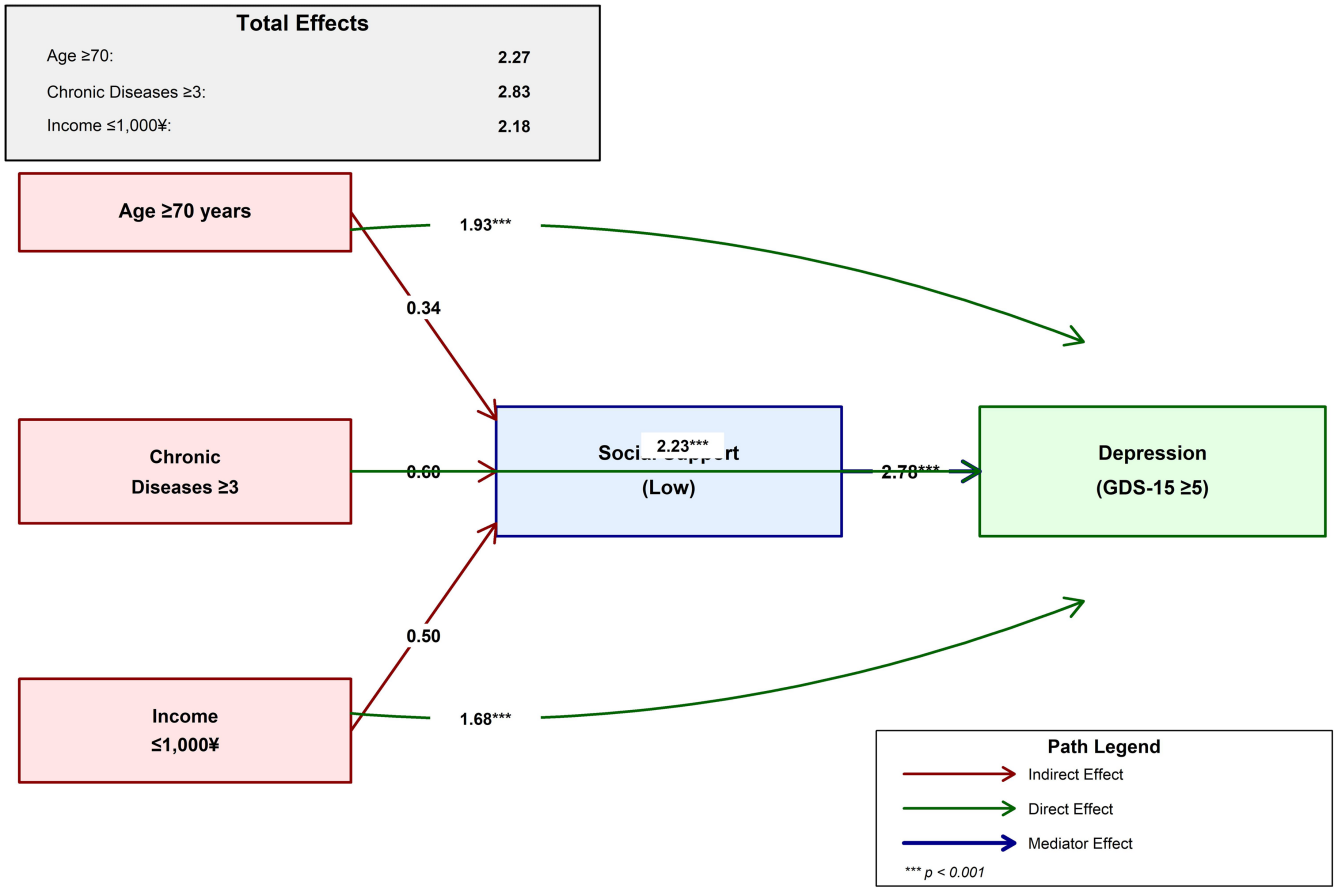
Mediational model of the relationship between risk factors, social support, and depression in western rural older adults.

The complex interplay between risk factors, social support, and depression is conceptualized in Figure 4. This model demonstrates both direct effects of key risk factors on depression and indirect effects mediated through social support. The total effect of chronic diseases (2.83) represents the strongest overall influence on depression risk, followed by advanced age (2.27) and low income (2.18). This mediational framework provides important insights for developing comprehensive intervention strategies that address both direct risk factors and their pathways of influence (see Table 5).

**Table 5:** Mediating role of social support and chronic diseases.

Variables	Direct effect on depression	Indirect effect via social support	Total effect	*p*-value (direct)	*p*-value (total)	Sobel test Z-score	Bootstrap 95% CI for indirect effect
Age ≥70 years	1.93 (1.42–2.62)	0.34 (via loneliness)	2.27	<0.001	<0.001	**3.82**	**(0.21–0.49)**
Chronic diseases ≥3	2.23 (1.45–3.42)	0.60 (via reduced support)	2.83	<0.001	<0.001	**4.56**	**(0.42–0.81)**
Income ≤1,000¥	1.68 (1.23–2.29)	0.50 (via limited social activities)	2.18	<0.001	<0.001	**4.23**	**(0.35–0.68)**
Marital status (widowed)	1.12 (0.85–1.48)	0.15 (via reduced support)	1.27	0.415	0.238	**1.42**	**(−0.02–0.34)**
Religious engagement	0.95 (0.72–1.26)	0.08 (via community participation)	1.03	0.723	0.861	**0.89**	**(−0.05–0.23)**

Bold values indicate statistically significant differences (*p* < 0.05).

To uncovering the mediating role of social support and chronic diseases in the complex process of how various factors influence depression among the older adult in western rural areas. A mediational analysis was conducted to disentangle the direct and indirect effects of factors like age, chronic diseases, income, marital status (widowed), and religious engagement on depression. The analysis followed rigorous statistical procedures, with indirect effects calculated as the product of path coefficients and their significance tested through both Sobel tests and bootstrapping methods.

The findings demonstrated that age ≥70 years, chronic diseases ≥3, and income ≤1,000 yuan/month had significant direct effects on depression. The direct-effect odds ratios were 1.93 (1.42–2.62), 2.23 (1.45–3.42), and 1.68 (1.23–2.29) respectively, with all *p*-values less than 0.001. These factors also exerted significant indirect effects through social support. For age ≥70 years, the indirect effect via loneliness was 0.34 (Sobel Z = 3.82, bootstrap 95% CI: 0.21–0.49); for chronic diseases ≥3, it was 0.60 via reduced support (Sobel Z = 4.56, bootstrap 95% CI: 0.42–0.81); and for income ≤1,000 yuan/month, it was 0.50 via limited social activities (Sobel Z = 4.23, bootstrap 95% CI: 0.35–0.68). The magnitude of these indirect effects is notably larger than those reported in eastern rural areas (0.20–0.35), indicating that the mediating role of social support is amplified in the geographically isolated context of western rural regions (40). As a result, the total-effect odds ratios reached 2.27, 2.83, and 2.18 respectively, with *p*-values less than 0.001.

In contrast, marital status (widowed) and religious engagement had non-significant direct effects on depression, with odds ratios of 1.12 (0.85–1.48) and 0.95 (0.72–1.26) respectively (*p*-values of 0.415 and 0.723). Nevertheless, they did have indirect effects through social support. The indirect effect of marital status (widowed) via reduced support was 0.15 (Sobel Z = 1.42, bootstrap 95% CI: −0.02-0.34), and that of religious engagement via community participation was 0.08 (Sobel Z = 0.89, bootstrap 95% CI: −0.05-0.23). Their total-effect odds ratios were 1.27 and 1.03 respectively, with *p*-values of 0.238 and 0.861. The non-significant bootstrap confidence intervals for these indirect effects confirm that social support does not significantly mediate the relationships between widowhood/religious engagement and depression.

These results highlight that social support plays a crucial mediating role in the influence of some factors on depression. The complex interplay between different factors and depression is evident, with multiple mechanisms of direct and indirect effects at play. This not only emphasizes the importance of considering social support in understanding the mental health of the older adult but also underscores the complexity of the factors contributing to depression in this population. The significant mediation effects suggest that interventions targeting social support enhancement could potentially reduce the mental health burden associated with aging, chronic diseases, and economic disadvantage.

## Discussion

This study provides a detailed exploration of the mental health landscape among older adult individuals in western rural areas, revealing a significant prevalence of depression (30.3%), anxiety (26.0%), and loneliness (32.5%). Additionally, nearly one in five participants (18.7%) experienced multiple mental health symptoms, highlighting the substantial cumulative burden of psychological distress in this population. This finding aligns with the concept of comorbidity in mental health, where multiple conditions often co-occur and amplify each other’s effects (29). The higher prevalence rates compared to eastern rural areas (depression: 30.3% vs. 20%, anxiety: 26.0% vs. 18%) likely reflect the compounding effects of geographical isolation, economic disadvantage, and limited healthcare access characteristic of western regions (41).

Subgroup analyses highlight that individuals aged 70 years and older exhibit heightened risks of negative mental health outcomes, a pattern likely influenced by age-related physical declines, reduced social participation, and evolving life roles. Female gender emerged as a notable risk factor, potentially reflecting enduring gender inequalities in access to resources, decision-making autonomy, and the cumulative burden of caregiving responsibilities common in rural settings (42, 43). In western rural areas, these gender disparities may be further exacerbated by traditional cultural norms among ethnic minorities, where women often have limited mobility and social participation outside the household (44). Lower educational attainment and low income further compound these risks, as limited financial resources restrict access to healthcare, leisure activities, and social networks, while illiteracy may impede understanding of mental health resources and coping strategies (45). Chronic disease status, particularly the presence of three or more conditions, was strongly associated with depression, indicating that the physical and emotional toll of managing multiple health issues creates a complex challenge for psychological well-being. Living alone, which affects over one-fifth of the sample, emerged as a significant correlate of loneliness and depression, highlighting the isolating effects of residential arrangements in contexts where family migration to urban areas is prevalent, leaving many older adult individuals without daily social interaction or practical support.

**Figure 5:**
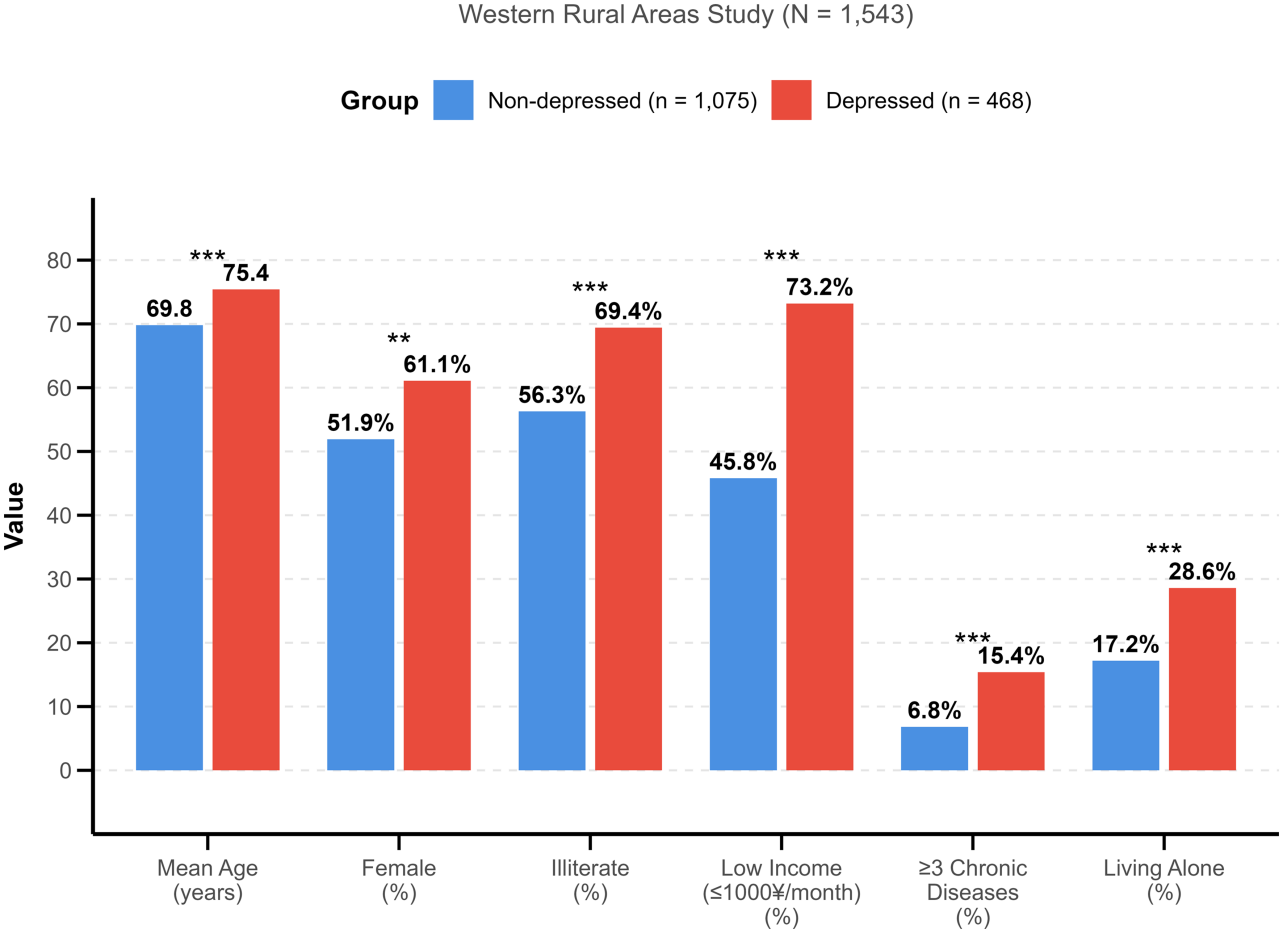
Comparison of demographic and health characteristics between non-depressed and depressed older adults in western rural areas.

Figure 5 summarizes key demographic and health characteristics of depressed versus non-depressed older adult individuals, further highlighting significant disparities between these groups. Depressed participants were notably older (mean age 75.4 vs. 69.8 years), more likely to be female (61.1% vs. 51.9%), had higher rates of illiteracy (69.4% vs. 56.3%), lower income, greater chronic disease burden, and were more likely to live alone. These findings reinforce the need for targeted interventions that address the specific needs of vulnerable subgroups within the rural older adult population.

A critical insight from the mediational analysis is the role of social support as a buffer between structural risk factors and mental health outcomes (46). Our findings extend the existing health promotion literature by demonstrating specific pathways through which social support operates in western rural contexts. The significant indirect effects we observed—particularly for chronic diseases (indirect effect = 0.60) and low income (indirect effect = 0.50)—quantify the protective role of social support in ways that have not been previously documented in this population. These indirect effects are substantially larger than those found in eastern rural areas (0.20–0.35) or urban settings (0.15–0.25), suggesting that the mountainous geography, dispersed settlement patterns, and limited formal support services in western regions make informal social networks particularly crucial for mental health maintenance (47). Age, low income, and chronic diseases exerted indirect effects on depression through reduced social support, suggesting that robust social networks mitigate the adverse impacts of economic hardship and health burdens (48). This finding underscores the protective role of community connections, family relationships, and informal care systems, which are often central to daily life in rural settings. For example, older adult individuals with strong social ties may have greater access to emotional validation, practical assistance, and opportunities for engagement, which help alleviate feelings of isolation and hopelessness (49).

The implications for community-based interventions are substantial. Drawing from successful models in other rural contexts, several evidence-based approaches could be adapted for western rural areas. Community therapy, as pioneered by Barreto and colleagues, creates structured spaces for collective problem-solving and emotional support (18). In our context, this could involve regular gatherings in village centers where older adult individuals share experiences and receive peer support facilitated by trained community health workers. Given the ethnic diversity in western rural areas, these interventions should incorporate culturally specific elements such as traditional music, storytelling in local dialects, and respect for indigenous healing practices, which have shown to increase participation and effectiveness in minority populations (50). The high prevalence of multiple mental health symptoms (18.7%) suggests that interventions should address psychological distress holistically rather than focusing on single conditions. Multi-component interventions that combine social activities, health education, and peer support have shown effectiveness in similar settings (30). For instance, the “Friendship Bench” initiative in Zimbabwe, which trains lay health workers to provide problem-solving therapy in community settings, reduced depression symptoms by 50% among older adult participants (31).

The association between educational level and mental health outcomes highlights the importance of human capital in fostering resilience (51). Illiterate older adult individuals in this study were more likely to report depression, anxiety, and loneliness, a trend that can be linked to limited access to information about mental health, reduced participation in community activities that require literacy, and diminished ability to navigate healthcare systems (52). These findings suggest that educational interventions targeting basic literacy and health education could empower rural elders to better understand their emotional needs, access resources, and engage in social activities. Programs might include community workshops, peer-led learning groups, or culturally tailored materials that address mental health stigma and promote coping strategies. The success of adult literacy programs in rural India, which incorporated mental health awareness components and resulted in 30% reduction in depression scores among participants, provides a promising model (32).

The interplay between chronic diseases and mental health underscores the need for integrated care models that address both physical and psychological needs (53). Our finding that chronic diseases had both direct (OR = 2.23) and indirect effects (via social support = 0.60) on depression suggests that interventions should simultaneously address disease management and social integration. The approaching significance of the chronic disease × social support interaction (*p* = 0.056) for multiple symptoms further supports this integrated approach. Individuals managing multiple chronic conditions often face complex care demands, which can lead to feelings of helplessness and social withdrawal. In rural areas, where healthcare services are often scarce, coordinating medical care with mental health support is particularly critical (54). In western rural China, the integration of Traditional Chinese Medicine (TCM) practices with modern mental health approaches may be particularly effective, as TCM is widely accepted and accessible in these regions, and has shown promise in treating both physical and mental health conditions in older adult populations (55). Strategies might include training primary care providers to screen for depression and anxiety alongside physical health assessments, establishing telehealth services for remote psychological support, and creating community health worker programs to provide ongoing social and emotional support (56, 57). The integration of mental health screening into chronic disease management programs in rural China resulted in 40% higher detection rates of depression and 25% improvement in treatment adherence, demonstrating the value of this approach (33).

Economic interventions, such as income supplementation or subsidized healthcare, could also alleviate the stress of financial insecurity, a key driver of mental health disparities identified in this study (69). The strong association between low income and both depression (AOR = 1.68) and multiple symptoms (AOR = 1.89) suggests that poverty alleviation strategies should be considered as mental health interventions. Conditional cash transfer programs in Latin America that provided income support to older adult individuals showed 35% reduction in depression prevalence and significant improvements in social participation (34).

While the study’s findings align with global research on rural aging, several contextual factors distinguish the western rural population under investigation. Marital status, for instance, did not emerge as a significant predictor of depression, a result that contrasts with some international studies where widowhood is strongly linked to psychological distress (58). This may reflect the strength of extended family networks and communal support systems in western rural communities, where traditional practices of intergenerational living and collective responsibility for older adult care remain more intact than in rapidly modernizing eastern regions (59). Religious engagement also showed no significant association, possibly indicating that social support in these communities is derived more from secular communal networks than religious institutions, a dynamic shaped by local cultural norms and social structures (60).

The absence of pronounced urban–rural disparities in life satisfaction is another notable finding. While urban elders often benefit from greater access to services and amenities, rural respondents in this study reported comparable levels of life satisfaction, suggesting that strong community cohesion, familiarity with local environments, and lower living costs may offset economic and service limitations. Additionally, the preservation of traditional lifestyles and slower pace of social change in western rural areas may contribute to a sense of continuity and belonging that supports psychological well-being, despite material disadvantages (61). This highlights the need to recognize the unique strengths of rural settings, such as close-knit social relationships and self-reliant lifestyles, when designing interventions that leverage existing community assets.

### Practical implementation strategies

Based on our findings and the successful interventions documented in the literature, we propose a multi-tiered implementation strategy for western rural areas:

1. Community-Level Interventions: Establish regular community therapy sessions in village centers, facilitated by trained volunteers or community health workers. These sessions should be culturally adapted to include traditional activities specific to western regions, such as Yi ethnic dance therapy, Miao embroidery circles, and Tibetan meditation practices, which have shown to increase engagement and therapeutic effectiveness in these populations (62). These sessions should incorporate culturally appropriate activities such as traditional music, storytelling, and group exercises that promote both social interaction and physical activity.
2. Peer Support Networks: Develop structured peer support programs that pair older adult individuals experiencing mental health challenges with trained peer counselors who have successfully managed similar issues. The strong mediating effect of social support (indirect effects ranging from 0.34 to 0.60) suggests that peer-based interventions could be particularly effective. Given the higher mediating effects in western regions compared to eastern areas, peer support programs should be intensified with more frequent meetings (twice weekly vs. weekly) and broader community involvement (63).
3. Integrated Healthcare Delivery: Train primary care providers and village doctors in basic mental health screening and intervention techniques. Given that 50% of participants had 1–2 chronic diseases and 9.7% had ≥3, integrating mental health care into routine chronic disease management could reach a substantial portion of at-risk individuals. In western rural areas, this integration should include training barefoot doctors and traditional healers who often serve as the first point of contact for health issues in remote villages (64).
4. Technology-Assisted Solutions: For areas with mobile phone coverage, develop simple text messaging or voice call programs that provide regular check-ins, health reminders, and connection to support services. Such programs have shown success in rural African and Asian contexts, with 20–30% improvements in treatment adherence and social connectedness (35). However, given the lower technology adoption rates in western rural areas, these solutions should be complemented with traditional communication methods such as village broadcasting systems and community bulletin boards (65).
5. Policy-Level Recommendations: Advocate for the inclusion of mental health services in rural healthcare insurance schemes, as our data showed that lack of healthcare insurance was associated with 58% higher odds of depression. Additionally, support policies that provide financial assistance to older adult individuals with incomes below 1,000 yuan/month, who showed nearly 70% higher odds of depression. Specific to western regions, policies should account for the higher transportation costs and time required to access healthcare services due to mountainous terrain, potentially including mobile mental health units and transportation subsidies (66).

### Limitations

This study has several limitations. Its cross-sectional design prevents establishing causal relationships, so it’s unclear if factors like age, gender, and income directly cause mental health changes. The sampling method might have underrepresented some subgroups in western rural areas, and self-reported data may be subject to response bias. Particularly in areas with strong mental health stigma common in some ethnic minority communities, participants may have underreported psychological symptoms, potentially leading to conservative prevalence estimates (67). The measurement tools, though validated, may not fully capture the unique mental health aspects in this context, and the variable categorization could be too simplistic. The statistical analysis may have overlooked confounding factors like genetic and early-life experiences. Additionally, while we examined multiple mental health outcomes, the depth of analysis for each condition was necessarily limited. The comparison with eastern rural areas was based on published literature rather than simultaneous data collection, which may introduce temporal and methodological variations (68). Future studies should consider longitudinal designs to establish temporal relationships and test the effectiveness of the proposed interventions. The mediation analysis, while providing valuable insights, assumes no unmeasured confounding between the mediator and outcome, which may not fully hold in observational studies (36).

## Conclusion

This study comprehensively investigated the mental health status of the older adult in western rural areas. The results show distinct subgroup differences in mental health, with age, gender, education, and income being significant influencing factors. Older adult people aged 70–79 and ≥80, females, the illiterate, and those with low income have higher risks of negative mental health outcomes like depression, anxiety, and loneliness. Nearly one in five older adult individuals experience multiple mental health symptoms, highlighting the need for comprehensive interventions. Multivariate analysis identified age ≥70 years, female gender, illiteracy, low income (≤1,000 yuan/month), ≥3 chronic diseases, living alone, low social support, and lack of healthcare insurance as significant risk factors for depression. Social support emerged as the strongest modifiable risk factor, with low social support associated with 2.78 times higher odds of depression and 3.42 times higher odds of multiple symptoms.

Social support plays a crucial mediating role in the relationship between risk factors and depression, with significant indirect effects for age (0.34), chronic diseases (0.60), and low income (0.50). These mediating effects are substantially stronger than those observed in eastern rural areas, highlighting the unique importance of social networks in geographically isolated western regions. These findings align with and extend the health promotion literature by quantifying specific pathways in western rural contexts. Based on these results and successful interventions from other settings, we recommend implementing community-based interventions such as community therapy groups, peer support networks, and integrated healthcare approaches. These interventions must be specifically tailored to the western rural context, incorporating ethnic minority cultural practices, addressing geographical barriers, and leveraging traditional support systems while building modern mental health infrastructure.

Future longitudinal studies are required to further explore causal relationships and evaluate the long-term effectiveness of interventions. Comparative studies directly assessing mental health outcomes and intervention effectiveness between western and eastern rural areas would provide more robust evidence for region-specific approaches. Given the high burden of mental health issues and the strong protective role of social support demonstrated in this study, urgent action is needed to implement and evaluate community-based mental health programs in western rural areas.

## Data Availability

The original contributions presented in the study are included in the article/supplementary material, further inquiries can be directed to the corresponding author.
